# Cribado y diagnóstico prenatal de anomalías genéticas: recomendaciones de consenso SEGO, SEQC^ML^, AEDP

**DOI:** 10.1515/almed-2019-0040

**Published:** 2020-06-22

**Authors:** Belén Prieto-García, Begoña Adiego, Javier Suela, Inmaculada Martín, Belén Santacruz, Javier García-Planells, Mar Gil, Concepción González, Eva Barrenechea

**Affiliations:** Servicio de Bioquímica Cínica, Hospital Universitario Central de Asturias, Departamento Bioquímica y Biología Molecular, Universidad de Oviedo, Avda Roma s/n – 33010, Oviedo, España; Servicio de Ginecología y Obstetricia, Hospital de Móstoles, Universidad Juan Carlos I, Madrid, España; Laboratorio de Genómica, NIMGenetics, Madrid, España; Servicio de Análisis Clínicos, Hospital Son Espases, Palma de Mallorca, España; Servicio de Ginecología y Obstetricia, Hospital Universitario de Torrejón, Madrid, España; Instituto de Medicina Genómica, IMEGEN, Valencia, España; Servicio de Análisis Clínicos, Hospital Universitario Virgen de la Macarena, Sevilla, España; Servicio de Análisis Clínicos, Hospital Universitario de Galdakao, Galdakao, España

**Keywords:** ADN libre circulante, cribado combinado, pruebas invasivas, translucencia nucal

## Abstract

El objetivo de este trabajo es difundir las recomendaciones del consenso entre las sociedades científicas SEGO, SEQC^ML^ y AEDP sobre cribado y diagnóstico prenatal de anomalías genéticas, así como una propuesta de indicadores de evaluación, desde una perspectiva de mejora de cada uno de los procesos que constituyen el campo de aplicación de las estrategias actuales de cribado: bioquímico, ecográfico y genético. Asimismo, se recogen recomendaciones relacionadas con los procesos invasivos de diagnóstico prenatal, incluyendo tanto las técnicas de recogida de muestra como las aplicables a su posterior análisis genético. *Perspectiva*: Se propone unificar criterios e indicadores a nivel nacional, con evaluaciones periódicas. Asimismo, sería muy recomendable establecer una estrategia de cribado prenatal a nivel nacional, dotada con recursos que aseguren la auditoría periódica de dichos indicadores y de los procedimientos diagnósticos, con supervisión por las administraciones sanitarias. Los protocolos deberían ser revisados periódicamente para adaptarse a nuevas tecnologías coste-efectivas.

## PARTE I. Cribado de anomalías genéticas en el primer trimestre

## 1 Proceso bioquímico

### 1.1 Recomendaciones preanalíticas

#### 1.1.1 Información para la embarazada y a los profesionales

Las gestantes deben ser informadas de los beneficios y las limitaciones del cribado prenatal de aneuploidías, obteniendo su consentimiento informado [1].

El profesional que realice la captación de la gestante recabará dicho consentimiento, generalmente verbal, informando sobre:

los derechos de la paciente
en qué consiste el cribado, qué pruebas incluye y su carácter voluntario
las opciones de actuación clínica ante un riesgo alto

Por su parte, el especialista del laboratorio clínico debe establecer:

los requerimientos para la correcta toma de muestra y sus condiciones de transporte
los datos necesarios para la solicitud e interpretación de las pruebas bioquímicas
los criterios preanalíticos de rechazo de muestras
otras informaciones de interés (tiempo de respuesta, modo de envío de resultados … )

#### 1.1.2 Datos del embarazo

Junto a los datos identificativos y la fecha de extracción, el facultativo del laboratorio necesita conocer la edad y peso de la embarazada y la edad gestacional estimada, para identificar valores críticos en los marcadores bioquímicos y gestionar la adecuada captación.

Además, deben introducirse en la aplicación para el cálculo del riesgo diversos factores correctores como: número de fetos y corionicidad (en embarazo gemelar), etnia, técnicas de reproducción asistida (TRA), diabetes insulinodependiente, tabaquismo y antecedentes de embarazo anterior con aneuploidía.

#### 1.1.3 Toma de muestra

La muestra de sangre, obtenida por flebotomía, se identificará de forma inequívoca, al menos con dos identificadores propios.

#### 1.1.4 Estabilidad y transporte

Se recomienda separar el suero y conservarlo a 4 °C hasta su análisis, que se realizará preferiblemente en las primeras 72 horas desde su recogida. Para almacenamientos de mayor duración, sobre todo si la entrega de muestras supera el plazo recomendable (≤24 h), el suero se congelará a −20 °C, evitando ciclos de congelación-descongelación [2].

#### 1.1.5 Custodia

Dada la estabilidad de los marcadores, se recomienda conservar a −20 °C una alícuota de las muestras durante un año, para posibles reclamaciones o verificaciones de resultados.

Según la Norma UNE-EN ISO 15189:2013, los protocolos analíticos, registros de muestras y análisis (incluyendo calibraciones, control de calidad, resultados, etc.), así como los registros de competencia y calidad del laboratorio para la medida de los marcadores, deben conservarse durante 5 años.

### 1.2 Recomendaciones analíticas

#### 1.2.1 Metodología y reactivos

Los reactivos para la determinación sérica de los marcadores bioquímicos, proteína A plasmática asociada al embarazo (PAPP-A) y fracción beta libre de la gonadotropina coriónica humana (βhCG libre), deben poseer el marcado de conformidad europea (CE) para cribado de Síndrome de Down o trisomía 21 (T21), con experiencia demostrable y medianas calculadas según la edad gestacional. La estabilidad de los reactivos durante largos periodos de tiempo reduce el impacto de las diferencias entre lotes de fabricación.

Existen protocolos de certificación basados en los criterios mínimos que deben cumplir las plataformas analíticas, los reactivos y los programas de cálculo de riesgo prenatal [2], [3], [4]. En la actualidad, el algoritmo de cálculo de la *Fetal Medicine Foundation* es compatible con todas las plataformas analíticas disponibles excepto DPC Immulite 2000 [5].

Se recomienda utilizar plataformas con la menor imprecisión de medida, para asegurar una imprecisión del 10% en los puntos de decisión clínica (riesgo 1/250 o 1/270) [6].

#### 1.2.2 Estándares de calibración

Se recomienda utilizar reactivos fabricados según la directiva europea IVD 98/79/EC y las normas ISO17511:2003, estandarizados frente a patrones internacionales con trazabilidad a los patrones WHO IRP 75/551 y WHO RR 99/650 para βhCG libre y al patrón WHO IRP 78/610 para PAPP-A. Se recomienda la expresión de los resultados en UI/L o en mUI/mL.

#### 1.2.3 Aseguramiento de la calidad analítica

Es esencial utilizar controles internos de calidad validados, a ser posible de diferente proveedor que el del ensayo utilizado, con estabilidad y caducidades suficientemente amplias para poder evaluar y minimizar la variabilidad entre lotes de reactivos.

El protocolo de calidad analítica incluirá:

tipo y frecuencia de medida de los controles
límites de tolerancia
protocolo de calibración y medidas correctoras

Con cada serie analítica, se recomienda medir controles a tres concentraciones diferentes para cada marcador, acordes a las esperadas en el periodo gestacional correspondiente. El estándar de imprecisión óptima se alcanza con coeficientes de variación interdiarios inferiores a 3,5% [5].

En Europa, el programa de intercomparación más extendido para cribado prenatal, UK-NEQAS, analiza los datos obtenidos mensualmente y aporta un informe anual, incluyendo tanto el estudio del sesgo y la imprecisión de los marcadores bioquímicos, como una valoración del riesgo estimado en cada muestra [4], [7].

#### 1.2.4 Conversión de los resultados en múltiplos de la mediana (MdM)

Los resultados deben expresarse en MdM según la edad gestacional y aplicar los factores correctores indicados en el apartado 1.1.2, que influyen significativamente en los MdM de los marcadores bioquímicos [8] y en el cálculo del riesgo. Por tanto, deben proporcionarse desde la solicitud del cribado [9] y cada laboratorio debe auditarlos periódicamente, realizando ajustes locales si fuese necesario [4].

### 1.3 Recomendaciones postanalíticas

#### 1.3.1 Programa de cálculo del riesgo

Se deben exigir unas especificaciones mínimas a las aplicaciones informáticas que calculan el riesgo (Tabla 1), dada la amplia variabilidad de resultados según el programa aplicado [10].

**Tabla 1:** Recomendaciones para el programa de cálculo del riesgo.

– Ajuste de los marcadores por los factores de corrección necesarios – Flexibilidad para actualizar las variaciones locales en los parámetros de distribución y peso materno, así como para incluir nuevos marcadores y factores de corrección – Posibilidad de utilizar diferentes curvas de edad – Posibilidad de corrección por gestaciones previas con T21 – Expresión del riesgo a término o en el momento del análisis – Identificación de los modelos de los marcadores definidos para las aneuploidías más frecuentes – Facilidad para el cálculo de los indicadores de calidad – Posibilidad de introducción de los resultados de las pruebas con el ADNlc y las pruebas invasivas, así como los posibles desenlaces (incluyendo las muertes intrauterinas y perinatales, interrupciones del embarazo y abortos espontáneos) – Facilidad para exportar los datos para las auditorías regionales o nacionales – Certificación CE, obligada desde 2005, según la directiva 98/79/CE y Real Decreto 1662/2000 sobre productos sanitarios para el diagnóstico “*in vitro*”

El programa debe permitir la definición de diferentes poblaciones de embarazadas. En la población no afecta, la mediana de los MdM debe ser de 1,00. Como la desviación estándar poblacional depende del método de cribado, es aconsejable que cada laboratorio utilice sus propias desviaciones para cada marcador. Para ello debe disponer de más de 1.000 cribados. En el primer trimestre del embarazo, las desviaciones deben situarse entre los siguientes límites: para βhCG libre, de 0,25 a 0,29 y, para PAPP-A, de 0,23 a 0,29. Si los valores están fuera de estos límites, se investigarán los motivos para emprender las acciones correctivas pertinentes.

Para las gestaciones portadoras de fetos afectos de trisomías, la mediana y la desviación estándar se obtendrán de publicaciones con gran número de casos y el programa debe actualizarse periódicamente.

Dado que no son totalmente independientes, se recomienda que el laboratorio calcule los coeficientes de correlación entre cada par de marcadores para su propia población, que deberían estar comprendidos entre 0,05 y 0,25 en el caso de PAPP-A con βhCG libre.

Es aconsejable truncar los valores extremos en MdM, con un límite inferior de 0,2 y superior de 5,0, tanto para βhCG libre como para PAPP-A. Los programas deben permitir modificar los truncajes cuando sea pertinente.

#### 1.3.2 Formato de entrega de resultados

El formato de envío de resultados debe adaptarse a las necesidades de la población gestante atendida [2], [11]. En general, se recomiendan plataformas online, con introducción de datos informatizada para cada proceso y cuyo software garantice la trazabilidad de los datos, con identificación inequívoca del usuario.

El informe para la gestante incluirá, al menos, los datos que se exponen en la Tabla 2 y será interpretado por el facultativo clínico responsable.

**Tabla 2:** Datos mínimos que debe incluir el informe del cribado combinado.

– Nombre de la embarazada, fecha de nacimiento y otro identificador único (número de historia clínica o de la seguridad social) – Nombre del facultativo peticionario y centro al que pertenece – El cribado solicitado – Tipo de espécimen y la fecha en la que se ha obtenido – Número de acceso de laboratorio que identifica la muestra – Datos demográficos e información utilizada en la interpretación (ej, LCR, edad y peso materno) – Medida de la TN y sus unidades interpretativas (ej: TN en mm) – Nombre y número credencial del ecografista – Resultados analíticos en unidades de masa (ej, ng/mL) y en unidades interpretativas (ej, MdM) ajustada por los factores de corrección – Resultado de riesgo para cada trisomía cribada

#### 1.3.3 Monitorización de los MdM

Las medianas proporcionadas por los fabricantes son útiles hasta disponer de medianas poblacionales propias, cuyo cálculo requiere un mínimo de 100–150 muestras en cada semana de gestación. Cada laboratorio debe supervisar sus medianas poblacionales con la periodicidad más adecuada a su actividad asistencial. El seguimiento de las medianas de los MdM permitirá verificar que se desvían +/−10% en torno a la unidad. En caso de detectar sesgos, permitirá anticipar acciones correctivas mediante una actualización de dichas medianas.

Las recomendaciones del proceso bioquímico se resumen en la Tabla 3.

**Tabla 3:** Resumen de recomendaciones del proceso bioquímico.

– La datación gestacional, el peso y la edad materna, la etnia, el hábito de fumar, la condición de insulino-dependencia y el embarazo por TRA (con edad de la donante, si procede) deben informarse al especialista del laboratorio para que pueda realizar una correcta valoración de las pruebas bioquímicas – La extracción de sangre debe realizarse por flebotomía convencional en la edad gestacional adecuada – El transporte y conservación de la muestra de suero se debe realizar a 4 °C siempre que vaya a ser entregada en las primeras 72 h desde la extracción. Para demoras superiores, se debe congelar a −20 °C hasta el envío – Se deben minimizar los ciclos de congelación-descongelación del suero – El laboratorio debe garantizar las condiciones preanalíticas y analíticas adecuadas para el cribado combinado. No se deben utilizar plataformas analíticas ni reactivos que no dispongan de marcado CE para este uso específico – Las prestaciones de error total deben ser verificadas mediante materiales de control de calidad interno y externo. Se recomienda participar en el programa de intercomparación UKNEQAS – Se recomienda la acreditación de las determinaciones bioquímicas necesarias para el cálculo del riesgo, por la norma UNE-EN ISO 15189:2013 – Deben utilizarse medianas ajustadas a la población gestante que se atiende para el cálculo de los MdM de cada marcador, con revisiones y actualizaciones periódicas. Para ello, se recomienda analizar más de 8.000 cribados anuales. No obstante, es aceptable un mínimo de 2.000 cribados anuales si se unifican varios laboratorios que atiendan a poblaciones similares, a la hora de evaluar las medianas de los marcadores bioquímicos – El software de cálculo debe poseer marcado CE para la estrategia de cribado realizada. Es responsabilidad del laboratorio conocer y verificar periódicamente las actualizaciones necesarias, tanto en los truncajes de los marcadores bioquímicos como en las diferentes curvas de correlación con la edad gestacional y con el peso materno – La transmisión informatizada de los resultados bioquímicos minimiza los errores de transcripción. Se debe garantizar la trazabilidad de cualquier cambio realizado, con identificación inequívoca de los profesionales que acceden al programa

## 2 Proceso ecográfico: control de calidad de los parámetros ecográficos del cribado combinado de primer trimestre

Los dos parámetros ecográficos considerados para el cribado combinado del primer trimestre son la longitud cráneo raquis (LCR) y la traslucencia nucal (TN).

### 2.1 Condiciones técnicas y asistenciales

Para la adecuada medición de la LCR y la TN deben cumplirse una serie de condiciones que afectan al equipo de ultrasonografía empleado (resolución de gama media-alta), el tiempo dedicado a la exploración (al menos 25 minutos), la metodología empleada y la capacitación del explorador, que poseerá formación específica en el estudio del primer trimestre [12], [13].

### 2.2 Formación y acreditación

El estudio ecográfico del primer trimestre debe ser realizado por especialistas con experiencia y formación adecuadas. En España, dicha formación está recogida en el programa formativo de los médicos internos residentes. La Sociedad Española de Ginecología y Obstetricia (SEGO) otorga el nivel de capacitación ecográfica especializada para aquellos ginecólogos que se formen dentro de dicho programa en centros acreditados.

Desde hace años, tanto la Sección de Ecografía de SEGO (SESEGO) como otras entidades, desarrollan periódicamente diversas actividades específicas en este tema, que han posibilitado la difusión y el aprendizaje a los especialistas. En 2018, se publicó la Guía de Asistencia Práctica de cribado del primer trimestre en el que se aconseja realizar un control de calidad del programa de cribado [14].

No existe ninguna entidad en España que acredite ni audite los criterios de calidad ecográficos del cribado del primer trimestre. Algunas comunidades autónomas tienen programas de control de calidad del cribado del primer trimestre, pero otras muchas no han desarrollado estos aspectos.

En este documento, se exponen los procedimientos de calidad recogidos en las guías internacionales europeas y americanas, instauradas en otros países de nuestro entorno.

En Europa, desde 1992, la *Fetal Medicine Foundation* desarrolla una exhaustiva investigación en el estudio ecográfico del primer trimestre, proponiendo requisitos técnicos y estándares y un sistema de acreditación individual avalado por la UK-NEQAS. En EEUU, la Sociedad de Medicina Materno-Fetal ha creado el programa de formación *Nuchal Translucency Quality Review*, con similares objetivos [15].

### 2.3 Evaluación de la calidad de la medición de la LCR

#### 2.3.1 Criterios estándar para la medición de la LCR

**Figura 1:**
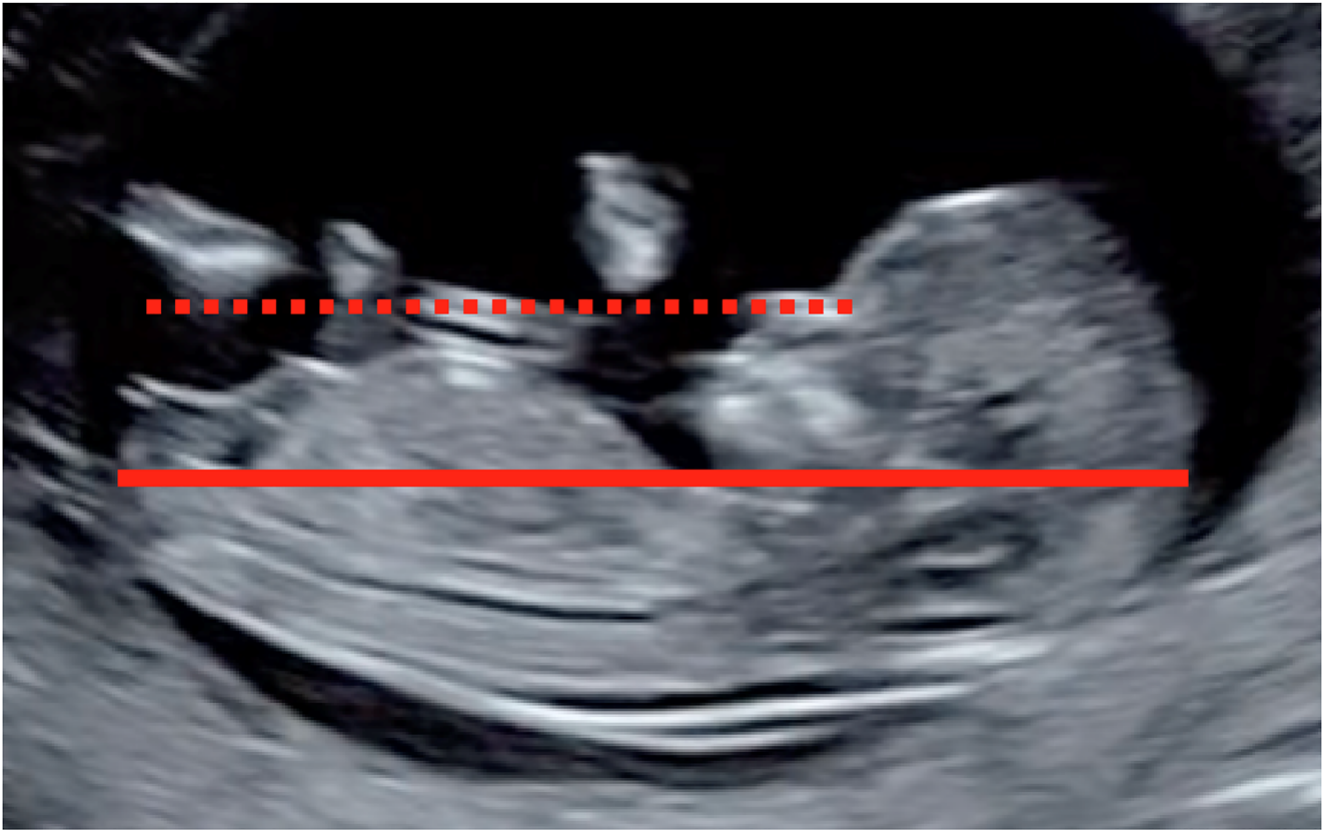
Criterios estándar para la medición de la LCR. La valoración puede realizarse por vía abdominal o vaginal. **LCR** entre 45 y 84 mm. **Plano mediosagital:** Sección sagital del feto con la cabeza alineada con el cuerpo. Debe visualizarse la punta ecogénica de la nariz, el hueso nasal si está presente, la forma rectangular del paladar, el diencéfalo, (no debe incluir la órbita), la inserción del cordón umbilical, la vejiga y el tubérculo genital. Los miembros inferiores no deben ser visibles. **Correcta visualización del polo cefálico y caudal,** con identificación de la coronilla, la rabadilla y la piel que los recubre. **Posición fetal** neutral, ni flexionada (debe identificarse una bolsa de líquido amniótico al menos equivalente a la anchura del paladar entre la barbilla y el tórax); ni extendida (ángulo del paladar entre 30–60° respecto a la horizontal). **Orientación**: el plano de la LCR debe estar entre 0–30° respecto a la horizontal de tal manera que la línea de medición de la LCR forme un ángulo de 90° con el haz de ultrasonidos. Una forma de asegurar la posición horizontal del feto es trazar una línea desde la punta de la nariz, que ha de estar al nivel o por encima de la pared abdominal en relación con la horizontal. **Magnificación**: la sección completa de la LCR debe ocupar más del 60% de la pantalla y debe incluirse en su totalidad. **Colocación correcta de los cálipers** cubriendo la máxima distancia entre el borde externo de coronilla y la rabadilla. Debe medirse al menos en 3 ocasiones y registrar la medida mayor que cumpla los criterios adecuados.

Es fundamental seguir los estándares internacionales y asegurar una estricta adherencia a los mismos [12], [16] como se detalla en la Figura 1.

#### 2.3.2 Métodos de control de calidad en la medición de la LCR

Mientras que la TN ha sido sometida a programas de control de calidad, sorprendentemente son raras las auditorías de la medida de la LCR y los programas de control de calidad están muy por detrás de los aplicados a la TN.

Recientemente se han propuesto métodos cualitativos basados en la evaluación de imágenes mediante puntuaciones (*Image Scoring Method*: ISM), análogos a los de la TN [17], [18], [19], que resultan útiles en el entrenamiento y acreditación, aunque no son aptos para realizar auditorías a gran escala.

La evaluación cuantitativa de la calidad de la medida de la LCR basada en la distribución de las medidas observadas es mucho más sencilla de implementar a gran escala, pero tiene el obstáculo de que no pueden utilizarse frente a referencias biométricas como se hace con la TN respecto a la LCR. Se ha propuesto utilizar como referencia las desviaciones específicas de los marcadores bioquímicos (PAPP-A y βhCG), que son producidas por desviaciones sistemáticas en la medida de la LCR [20].

#### 2.3.3 Evaluación de la calidad de la medición de la TN

##### 2.3.3.1 Criterios estándar para la medición de la TN

La TN es el componente de mayor potencia en el cálculo del riesgo de aneuploidía pero, debido a su importante dependencia del operador, está sometida a una gran variabilidad, muy superior a la de los marcadores bioquímicos, por lo que es imprescindible la adherencia a una técnica estandarizada.

Esto es trascendental porque mínimas desviaciones en la medida de la TN pueden causar importantes cambios en la eficacia del cribado. Como podría esperarse, la imprecisión en la medida cambia en el mismo sentido la tasa de detección (TD) y la de falsos positivos (FP). Una infraestimación reduce la TD del 70% al 63% y los FP del 2,7% al 1,2% y al contrario sucede con la sobreestimación [21].

**Figura 2:**
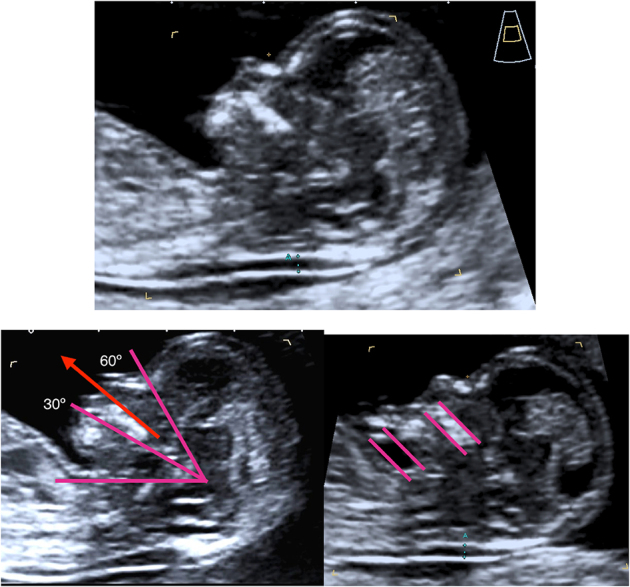
Criterios estándar para la medición de la TN. La valoración puede realizarse por vía abdominal o vaginal. **LCR** entre 45 y 84 mm. **Magnificación:** la imagen debe incluir solo la cabeza y parte superior del tórax. **Feto en posición neutra,** con la cabeza alineada con la columna (una posición flexionada puede disminuir falsamente la medida de la TN mientras que una posición en extensión puede aumentarla). Los criterios para asegurarnos una posición neutra de la cabeza son: (1) La línea del paladar debe estar entre 30 y 60º con la horizontal. (2) Debe haber un bolsillo de líquido amniótico equivalente al menos al grosor del maxilar entre la barbilla y el tórax. **Sección medio-sagital**, utilizando como marcadores que deben visualizarse en el mismo plano la punta ecogénica de la nariz, la forma rectangular del paladar, el diencéfalo y la TN. El proceso superior del maxilar superior no debe ser visible. Debe medirse la zona de **máxima** sonolucencia. **Calipers on-on:** el borde interno de la línea horizontal del cáliper debe colocarse encima de la línea que define la TN. **Reducir la ganancia** para evitar incluir en la medida la zona difuminada del límite de la TN. **Identificación de la membrana amniótica** separada del feto y de la posible interposición del cordón. En caso de la presencia de cordón umbilical alrededor del cuello fetal, debe tomarse la media de las medidas por encima y por debajo del cordón. **Medir al menos 3 veces** y elegir la mayor de tres medidas satisfactorias.

En la Figura 2 se muestran los criterios óptimos que se deben seguir para la medición correcta de la TN [12], [13], [22].

##### 2.3.3.2 Métodos de control de calidad en la medición de la TN

El control de calidad en la medición de la TN puede emplear criterios cualitativos o cuantitativos.

El método cualitativo se fundamenta en sistemas de puntuación ISM basados en la calificación por un equipo revisor de las imágenes, de acuerdo con determinados criterios [23], [24], [25]. Es especialmente útil durante el entrenamiento inicial, o cuando se necesita un reentrenamiento si se identifica una desviación respecto a la norma, pero su aplicación requiere mucho tiempo y es excesivamente costosa.

Por ello, en la práctica clínica, las recomendaciones de las sociedades internacionales se fundamentan en análisis cuantitativos [26] o en diagramas de gráficos, que son aplicables a gran escala.

El método propuesto por el WIHRI (*Women & Infants Hospital of Rhode Island*) ha sido implantado con éxito en el estudio multicéntrico FASTER [27], [28]. Es similar al que se realiza sobre los marcadores bioquímicos y consiste en el análisis de parámetros estadísticos como la mediana de MdM, la desviación estándar a escala logarítmica de los MdM (objetivo: 0,08–0,13), o el incremento porcentual por semana gestacional (objetivo 15–35%). La mediana de los MdM es el mejor estimador, ya que no está sujeto a la distorsión producida por los valores extremos y se considera adecuado un valor entre 0,9 y 1,1 MdM.

El inconveniente de estos métodos es que las desviaciones sólo se detectan retrospectivamente y por tanto el *feed-back* hacia el ecografista para corregir la técnica se retrasa inevitablemente.

Como alternativa o complemento se propone el método CUSUM (*cumulative sum)* [29], [30], [31], [32]. Este método se basa en que, en cualquier proceso médico, existe una cierta variabilidad natural alrededor de un valor establecido como diana que corresponde a la naturaleza del proceso y se considera normal. El método CUSUM calcula, cada vez que se hace una medición, el grado de desviación respecto a lo esperado y lo suma al resultado anterior (S+t y S−t). Cuanto más lejos está el valor observado del valor esperado, mayor es la suma acumulada y por lo tanto más se desvía el proceso del objetivo establecido.

El gráfico CUSUM es una representación de la tendencia de los resultados a lo largo del tiempo. Los sucesivos valores de S+t y S−t se representan gráficamente y se comparan frente a dos límites H+ y H− (superior e inferior). Si la curva muestra una pendiente y la línea de sobreestimación (o la de infraestimación) sobrepasan el límite de confianza, el proceso está fuera de control. Esta evaluación continua tiene la ventaja de ser prospectiva y permitir la detección temprana de las desviaciones.

##### 2.3.3.3 Comisiones de control de calidad

Sería aconsejable la creación de comisiones (locales, autonómicas o nacionales) para el control de calidad del cribado combinado del primer trimestre, para implementar un control de calidad en la medición de la TN y establecer el método de evaluación adecuado en cada entorno.

También es recomendable que exista un programa establecido para que los exploradores puedan recibir *feed-back* periódicamente de la distribución de sus mediciones. Es conveniente que las desviaciones significativas respecto a la distribución esperada que persisten en el tiempo sean informadas para identificar posibles causas.

## 3 Proceso genético

### 3.1 Recomendaciones preanalíticas

#### 3.1.1 Información para la embarazada y los profesionales

Aunque aplica la Ley 41/2002, previamente mencionada [1], según la Ley 14/2007, de 3 de julio, de Investigación biomédica, se recomienda utilizar un documento por escrito que incluya:

finalidad del análisis genético para el que consiente
lugar de realización del análisis y destino de la muestra biológica al término del mismo
personas que tendrán acceso a los datos de los análisis (cuando no vayan a ser anonimizados)
advertencia sobre la posibilidad de hallazgos inesperados, así como la facultad de decidir si se desea conocerlos
compromiso de suministrar consejo genético, una vez obtenidos los resultados

Por tanto, la gestante, en una consulta pre-test, debe dar su consentimiento escrito para la realización de la prueba, con conocimiento de las limitaciones del análisis, la interpretación futura del resultado y las pruebas complementarias necesarias.

El profesional prescriptor debe conocer toda la información correspondiente al test: cuándo está indicado, las alternativas diagnósticas posibles, los requerimientos preanalíticos de recogida, transporte y rechazo de muestras, así como la interpretación de los resultados y acciones posteriores.

#### 3.1.2 Datos del embarazo

Además de la información básica, descrita en el apartado 1.1.2, se requiere la indicación clínica, el valor del riesgo combinado, hallazgos ecográficos y antecedentes.

Es fundamental conocer las siguientes situaciones especiales, que hacen no recomendable el test o limitan su informatividad:

progenitor portador de translocación robertsoniana (indicar cuál)
índice de masa corporal >30 en la gestante [33]
exposición a heparina de bajo peso molecular [34]
embarazo por TRA, relevante en estudios de genotipado [35]
gemelo evanescente. No se recomienda un test de ADN libre circulante (ADNlc) con un gemelo evanescente
madre portadora de una patología a analizar (incluida en el test) [36]
transfusiones de sangre, transplante, infección generalizada o neoplasia en gestante, terapia de plasma: Todas estas circunstancias pueden alterar el resultado al introducir una cantidad indeterminada de ADN plasmático (endógeno o exógeno)

#### 3.1.3 Toma de muestra y condiciones de estabilidad y transporte

La sangre se recogerá por venopunción, identificando el tubo y el consentimiento/petición de manera inequívoca, al menos con dos identificadores propios. Se recomienda extraer un mínimo de 6 mL de sangre periférica con sistema de vacío, evitando la hemólisis y homogeneizando, sin agitación excesiva, el anticoagulante y la sangre mediante inversión del tubo.

Actualmente existen dos tipos de tubo de recogida útiles para analizar ADNlc (si bien su uso debería ser validado con el protocolo y la tecnología disponible antes de ser empleados en la rutina):

1. Tubo conteniendo anticoagulante EDTA. No contiene aditivos de preservación de ADNlc ni de prevención de rotura celular. No se recomienda este tipo de tubo si la muestra no se procesa entre las 4–8 horas desde su obtención. Tanto el transporte como la manipulación de la muestra deben realizarse a temperatura refrigerada (4 °C), evitando la congelación de la muestra.
2. Tubo de conservación de ADNlc. Son específicos para preservar el ADNlc durante períodos más prolongados, a la temperatura especificada por el fabricante. El tiempo máximo de utilidad tras la extracción debe ser validado analíticamente y fijado en la información suministrada al facultativo [37].

Toda muestra que no cumpla los requisitos de calidad indicados en la información preanalítica o que presente incidencias (coagulada, altamente hemolizada, etc.) no debe ser utilizada para el estudio del ADNlc.

#### 3.1.4 Custodia

La custodia de las muestras debe seguir los requerimientos generales de las normas para la certificación/acreditación de los laboratorios. Dada la estabilidad del plasma a −80 °C y de la librería genómica a −20 °C, se recomienda conservar en estas condiciones una alícuota de las muestras durante un año, para posibles reclamaciones o verificaciones de resultados.

### 3.2 Recomendaciones analíticas

#### 3.2.1 Metodología

Esta tecnología es de reciente implantación y no existe estandarización de sus criterios de calidad. Es fundamental que cada laboratorio especifique la metodología de trabajo, incluyendo la instrumentación, protocolo y personal técnico asociado. El test de ADNlc se puede aplicar de acuerdo a dos supuestos generales [38]:

cobertura: genoma completo a baja resolución o análisis de regiones específicas
sistema de análisis: método de conteo o método de genotipado

Debido a la variabilidad de tecnologías disponibles, no es posible una recomendación metodológica estricta. Se deben mantener condiciones ambientales y tecnológicas similares a las de los procedimientos de biología molecular para diagnóstico prenatal.

El algoritmo de análisis debería estar publicado y preferiblemente validado a nivel internacional, con datos concretos de la serie validada, tasa de verdaderos y falsos positivos, sensibilidad, especificidad, valores predictivos y tasa de no informatividad, entre otros aspectos. El algoritmo debe incluir una población suficiente, tanto de casos positivos para cada trisomía (T21, T18 y T13) como negativos, que certifique que el test ha sido probado en la población de análisis (tanto externa como local). Es recomendable disponer de estudios publicados con población general. En caso de que el algoritmo sea comercial, debe disponerse de los certificados de validación y acreditación correspondientes, así como de la idoneidad del algoritmo para la metodología utilizada.

Aunque no existe un consenso total, la mayoría de las guías sitúan el umbral de no informatividad en torno al 4% de fracción fetal (FF). Se recomienda que el algoritmo calcule la FF como control de calidad del test [39] y tener documentado el método de cálculo utilizado para estimarla, distinto de la exclusiva detección del cromosoma Y (ej: genotipado o cálculo de fragmentos), así como las limitaciones del mismo.

Los laboratorios que no realicen la técnica en sus instalaciones deben declarar dónde se realiza y, si fuera necesario, disponer de la documentación pertinente para su control.

#### 3.2.2 Aseguramiento de la calidad analítica

El laboratorio debe tener un sistema de validación procedimental, siendo recomendable realizar tanto control interno como externo. A nivel interno, puede utilizarse plasma de gestante (con resultado de trisomía o normalidad validado por una técnica invasiva) o plasma artificial suministrado por una compañía certificada. Se recomienda realizar un protocolo de validación anual para cada una de las situaciones a analizar (al menos, una determinación de T21, T18 y T13).

Se debe hacer seguimiento de los controles de calidad de protocolo, técnicos y materiales, así como controles ambientales para asegurar la máxima calidad.

Adicionalmente, se están desarrollando estudios de intercomparación realizados por entidades como GenQA (*Genomics Quality Assesment*, miembro de UKNEQAS). Es un esquema de carácter anual, con dos análisis realizados con la tecnología disponible en cada laboratorio y entrega de un informe. El estudio europeo del ADNlc para aneuploidías se encuentra en fase piloto, previendo que, en el año 2020, se convierta en un programa de intercomparaciones *de facto*, dada su implantación en los laboratorios europeos.

### 3.3 Recomendaciones postanalíticas

#### 3.3.1 Interpretación de los resultados

El estudio puede tener como resultados los siguientes: ALTO RIESGO para una de las trisomías analizadas, BAJO RIESGO para todas las trisomías analizadas y NO RESULTADO o resultado NO INFORMATIVO.

Este resultado debe ser siempre interpretado por un facultativo.

Un cribado con NO RESULTADO puede conducir a una demora diagnóstica debido a: repetición del test con el mismo material plasmático inicial, por un control de calidad insuficiente, o petición de nueva muestra por causas metodológicas, indicando la ventana recomendada para la nueva extracción (baja FF, otros). Por lo general, un resultado NO INFORMATIVO significa que no ha sido posible realizar un estudio adecuado en diferentes procesos/muestras. Deben recomendarse las acciones posteriores basándose en la evidencia clínica.

Existen algoritmos que combinan diferentes datos clínicos para calcular la razón de verosimilitud incluyendo el ADNlc. En este caso, el informe puede aportar un resultado de riesgo numérico, que deberá acompañarse de un valor umbral para riesgo alto o bajo.

Debe especificarse que un resultado de bajo riesgo no excluye absolutamente la posibilidad de un falso negativo, e interpretar siempre el resultado en el contexto de las demás pruebas clínicas. El resultado de bajo riesgo debe ir en consonancia con los valores predictivos negativos de cada trisomía.

Un resultado de alto riesgo debe acompañarse del valor predictivo positivo de la trisomía en cuestión. Se indicará, asimismo, que la muestra tiene bajo riesgo para el resto de trisomías. Todo resultado de alto riesgo debe ser confirmado mediante una técnica invasiva.

En el caso de que el test no pueda detectar triploidías completas, debe especificarse claramente en el consentimiento informado y en el informe correspondiente.

#### 3.3.2 Formato de entrega de los resultados

El informe de resultados debe incluir:

dos datos identificadores de la muestra y fecha de nacimiento
identificador interno del laboratorio
nombre del facultativo peticionario y centro de procedencia
tipo de muestra (sangre periférica, en este caso)
test solicitado y metodología, indicando algoritmo de uso
resultado del test en concepto de alto y bajo riesgo, acciones recomendadas a tomar
medida de FF (*cut-off*) y, si fuera relevante, porcentaje de cálculo
facultativo/s responsable/s del informe
fecha del informe
valores predictivos de la muestra indicando la población de estudio

El envío de resultados debe ser lo más seguro posible, recomendando interconexiones entre el software de gestión de laboratorio, portal de visualización con usuario y contraseña o envío por correo electrónico de un informe anonimizado y encriptado.

## PARTE II. Diagnóstico prenatal de anomalías genéticas mediante pruebas invasivas

## 1 Proceso ecográfico-obstétrico: control de calidad de las técnicas invasivas

Las estrategias actuales de cribado prenatal de aneuploidías requieren disponer de técnicas para obtener un diagnóstico definitivo, que permitan confirmar todo resultado de alto riesgo mediante pruebas genéticas sobre material fetal obtenido por técnica invasiva.

En este apartado se exponen los criterios de calidad que deben aplicarse a las técnicas invasivas derivadas del cribado combinado de cromosomopatías.

La práctica adecuada de técnicas invasivas exige habilidad adquirida mediante formación y experiencia. Está establecido que la tasa de procedimientos fallidos y pérdidas fetales se relaciona con la experiencia individual del operador [40], [41], [42] y con el número de procedimientos de cada centro [43].

En esta nueva era, dominada por el cribado mediante el ADNlc, el número de técnicas invasivas está disminuyendo dramáticamente y esto puede tener un considerable impacto en la formación y mantenimiento de la capacitación.

El entrenamiento debe adquirirse en el ámbito de centros avalados por las instituciones sanitarias. Para subsanar el impacto de la reducción de las técnicas invasivas, las instituciones deben plantearse la sustitución del modelo tradicional basado en el volumen, que se hará progresivamente insostenible, por soluciones novedosas basadas en la simulación o en la centralización de los procedimientos [44], [45], [46], [47] que han demostrado mejorar el aprendizaje y disminuir el número de intentos necesarios para completar el entrenamiento [48], [49].

No es posible exigir un número de procedimientos supervisados para optimizar los resultados y las cifras referidas en la literatura varían ampliamente entre 45 y 300 amniocentesis (AC) aunque no se espera mejora más allá de 100 procedimientos realizados independientemente [50], [51]. La curva de aprendizaje para la realización de una biopsia corial (BC) con el menor riesgo, se estabiliza a partir de los 175 procedimientos [52].

Tampoco es posible establecer en base a la evidencia científica el número mínimo de técnicas que deben realizarse al año para mantener la competencia una vez adquirida, aunque arbitrariamente, algunas instituciones lo han fijado en 30 [50].

Es imperativo disponer de un sistema de formación y auditoría no sólo de cada centro, sino sobre todo individual. Los resultados deben ser accesibles y transparentes tanto para los operadores como para los pacientes.

La monitorización de los resultados debe incluir una serie de parámetros para asegurar estándares de competencia y de calidad [50], [53]. Anualmente deberían revisarse los indicadores resumidos en la Tabla 4.

**Tabla 4:** Propuesta de registro para la evaluación anual de indicadores de los procedimientos invasivos.

– Número de procedimientos realizados – Pérdida gestacional en cualquier edad gestacional – Perdida gestacional a los 14 días del procedimiento – Pérdida gestacional antes de las 24 semanas – Número de punciones necesarias – Proporción de procedimientos que requieren más de una entrada – Proporción de procedimientos con muestra inadecuada o insuficiente – Proporción de amniocentesis indicadas tras BC por muestra inadecuada – Proporción de amniocentesis con muestra hemática – Tasa de fracaso de cultivo en técnicas citogéneticas tras BC o AC – Tasa de complicaciones: pérdida de líquido amniótico, parto pretérmino, infección, hemorragia, otras – Tasa de administración de profilaxis anti-D en mujeres RhD negativo

Se ha sugerido que la competencia debería ser reevaluada cuando la tasa de pérdida gestacional excede 4/100 o la tasa de fracaso supera la de 8/100 procedimientos consecutivos para la AC u 8/100 y 5/100 respectivamente para la BC [50].

Deberá considerarse la indicación de la técnica invasiva, porque en determinadas anomalías fetales es esperable una mayor tasa de abortos espontáneos no relacionados con el procedimiento. Independientemente del método de evaluación, el objetivo debe ser el seguimiento del 100% de los procedimientos; tasas inferiores probablemente subestimen la tasa de pérdida gestacional ya que estas tienden a concentrarse en las pérdidas de seguimiento [54].

Según el último estudio sistemático publicado [55], el riesgo ponderado de aborto tras una AC es de 0,91% (Intervalo de confianza 95%, IC 95%: 0,73–1,09%). El riesgo ponderado de pérdidas atribuibles a la AC es de 0,30% (IC 95%: 0,11–0,49%; I 2 = 70,1%).

El riesgo ponderado de aborto tras la realización de una BC es de 1,39% (IC 95%: 0,76–2,02%). El riesgo ponderado de pérdidas atribuibles al procedimiento de la BC es de 0,20% (IC 95%: −0,13–0,52%; I 2 = 52,7%).

## 2 Proceso genético

### 2.1 Consideraciones preanalíticas

#### 2.1.1 Tipo de muestra

Cualquier estudio del ADNlc con resultado de alto riesgo debería confirmarse sobre amniocitos libres en líquido amniótico, ya que este material es de origen exclusivamente fetal (a diferencia del ADNlc), lo que permite descartar la presencia de un mosaicismo de confinamiento placentario. No obstante, la BC puede ser una opción en determinado contexto clínico, dada la precocidad de la extracción, a pesar del riesgo de analizar material del mismo origen embrionario (trofoblasto). Por lo tanto, se empleará [56]:

Líquido amniótico, a partir de la semana 16 (nunca antes de la semana 15), para todas las circunstancias de confirmación. Serán necesarios 5–20 mL de líquido amniótico, contenidos en un tubo tipo Falcon (cónico) estéril. No se enviará al laboratorio la jeringa de extracción por el riesgo de pérdida del material durante el transporte
BC, a partir de la semana 11 (nunca antes de la 10), sólo para los casos de alto riesgo de T21. En casos de alto riesgo para T18 y T13 se requiere la presencia de marcadores ecográficos sugerentes del síndrome, debido a la posibilidad de un mosaicismo de confinamiento placentario. Se recomienda disponer, al menos, de 2 μL de material corial limpio, en un tubo tipo Eppendorf estéril, con un mínimo de 1 mL de suero fisiológico, tampón fosfato salino o medio de cultivo estéril para evitar la degradación del tejido. Para el cariotipo de BC se puede requerir más material de inicio.

Se recomienda disponer de fuente de ADN de la gestante (saliva, sangre) para descartar, si fuera necesario, contaminación materna en la muestra prenatal. Este estudio se recomienda siempre en caso de BC (al menos, para fetos femeninos) y cuando se analiza líquido amniótico hemático.

#### 2.1.2 Estabilidad y transporte

El transporte de la muestra requiere un sistema logístico que evite la rotura del tubo por aplastamiento (recomendable un recipiente rígido). Si el envío se realiza en menos de 24 horas, puede hacerse a temperatura ambiente. En caso contrario, es preferible el envío refrigerado (4–8 °C). Se recomienda no aceptar material fetal que haya superado un plazo de 72 horas desde su extracción, por el riesgo de disponer de un ADN no analizable. No se debe congelar el material.

### 2.2 Prueba genética indicada. Protocolo de priorización

La gestante debe conocer la técnica que se va a realizar en el laboratorio y firmar el correspondiente consentimiento informado.

#### 2.2.1 Técnicas rápidas (QF-PCR/FISH)

Inicialmente se recomienda la QF-PCR (PCR cuantitativa fluorescente) porque permite, en caso necesario, el estudio de la contaminación materna mediante una segunda QF-PCR en la muestra de la gestante. Se recomienda que el test cubra todas las aneuploidías generales: T13, T18, T21, X e Y, para confirmar, por una parte, el riesgo de cribado en las tres trisomías y por otra parte conocer el sexo fetal, realizándose el estudio de contaminación materna en caso de ser necesario. Por lo general, un resultado de confirmación mediante QF-PCR debería ser suficiente como metodología diagnóstica. El tiempo de respuesta recomendado será, como máximo, de 2 a 3 días laborables [57].

En el caso de que se sospeche un mosaicismo de bajo grado en el feto, se elegirá la técnica FISH (Hibridación *in situ* fluorescente), que es capaz de detectar mosaicismos cromosómicos por debajo del 20% de porcentaje celular. La QF-PCR puede detectar, como mucho, mosaicismos del 40%.

#### 2.2.2 *Microarrays* genómicos

Se recomienda utilizar un *array* orientado al diagnóstico prenatal [58], con un tiempo de respuesta máximo de 5 a 10 días laborables. Un resultado confirmatorio de la trisomía mediante *microarray* es considerado suficiente prueba diagnóstica, independientemente de los hallazgos ecográficos disponibles. En el caso de que no se confirme la trisomía señalada, se revisará el resto de las alteraciones analizables según el consentimiento informado correspondiente, sobre todo si la técnica puede detectar variantes de significado incierto. En cuanto a mosaicismos, la tasa de detección es de 20–30% para los *arrays* de hibridación genómica comparativa y de 10–20% para los *arrays* de polimorfismos de un único nucleótido.

#### 2.2.3 Cariotipo de bandeo G

La recomendación actual es combinar la QF-PCR y el cultivo citogenético largo (dos semanas de cultivo). En caso de no hacer QF-PCR, se realizará cultivo largo. Se recomienda realizar 2–3 cultivos independientes, al menos en dos incubadores separados, con diferentes condiciones y medios de cultivo, para prevenir la contaminación del material. Se recomienda informar del posible fallo del cultivo si se observa un comportamiento anómalo en los primeros 10 días naturales (7 días laborables), e informar finalmente del fallo en los siguientes 14 días naturales (10 días laborables). El 95% de los cariotipos debe ser informado en 10 días laborables.

El cariotipo de bandeo G para uso prenatal debe tener una resolución mínima desde 400 bandas para poder ser analizado, recomendando la revisión de un mínimo de 10 metafases (o superior en caso de un hallazgo genético en grado de mosaico).

#### 2.2.4 Protocolo de priorización

QF-PCR de primer orden

En BC, si el resultado es femenino y normal, se realizará QF-PCR de material materno. Si es positivo, será suficiente para dar resultado, pero si es negativo y no concordante con la ecografía, se recomendará una técnica de segundo orden.

*Microarray*/cariotipo en segundo orden

Para la detección de trisomías, ambas técnicas son igualmente útiles, teniendo el *microarray* la ventaja de su mayor rapidez y el cariotipo la detección de reordenamientos (translocación robertsoniana).

Consejo genético, posibilidad de estudio de progenitores

### 2.3 Control de calidad analítico

El laboratorio debe tener un sistema de validación procedimental, recomendable tanto interno como externo. Existen estudios de intercomparación realizados por entidades como GenQA. Son esquemas de carácter anual para cariotipo prenatal (en líquido amniótico y BC), QF-PCR para aneuploidías y *microarray* prenatal.

Se recomienda que el laboratorio emisor tenga experiencia contrastada en la técnica de elección para uso prenatal. Las tres técnicas deben tener un resultado analítico en >99% de las mediciones.

### 2.4 Interpretación de los resultados

Los resultados deben indicar DETECCION o NO DETECCION, con su interpretación clínica, ya que el estudio invasivo tiene carácter diagnóstico. Según las normas de buenas prácticas de los laboratorios de genética, debe recomendarse la comunicación en una consulta de consejo genético post test.

Debe informarse de las posibles repercusiones hereditarias. Por ejemplo, si se obtiene un resultado positivo en T21 y T13, es recomendable realizar un cariotipo fetal para detectar la posible presencia de una translocación robertsoniana y, en caso de positividad, un estudio de progenitores. El cariotipo fetal puede obviarse si se hace el cariotipo de los progenitores directamente para hacer un estudio de heredabilidad.

#### 2.4.1 Formato de entrega de los resultados

El informe debe incluir:

dos datos identificativos de la muestra y fecha de nacimiento
identificador interno del laboratorio
nombre del facultativo peticionario y centro de procedencia
tipo de muestra
test solicitado y metodología, indicando limitaciones
resultado del test (incluyendo fórmula citogenética por sistema internacional, ISCN)
interpretación del test y recomendaciones posteriores
facultativo/s responsable/s del informe
fecha del informe

El envío de resultados debe ser lo más seguro posible, recomendando dicho envío mediante interconexiones entre software de gestión de laboratorio, portal de visualización con usuario y contraseña o envío por correo electrónico de un informe anonimizado y encriptado.

### 2.5 Seguimiento de los resultados

Los resultados discordantes deben ser comunicados inmediatamente para realizar una auditoría del proceso, en la que debe estar disponible toda la documentación accesoria (sean falsos negativos o positivos). Por ello, una propuesta de los autores de este consenso es la creación de una base de datos nacional (o regional) en la que se incluyan todos los casos realizados en grupos hospitalarios públicos (y privados que quieran adscribirse), conteniendo la siguiente información:

identificador del test
fecha de registro y fecha de informe
indicación clínica, observaciones
test utilizado (especificar tipo: genoma completo/regiones particulares y método de conteo/genotipado/otro)
medición de la FF (SI/NO, %, método genotipado/tamaño fragmentos/otro)
modelo de producción: interno/externalizado
resultado del test (Bajo, T21, T18, T13)
confirmación analítica (BC/líquido amniótico, SI/NO)
hallazgos prenatales (por ejemplo, sospecha de falso negativo, aborto espontáneo, etc.)
fecha de parto
revisión del neonato: sano, afecto
resolución y explicación de incidencias

## Conclusiones

El presente documento consenso propone unificar criterios e indicadores de calidad por procesos (Material Suplementario, Tablas 1 a 3) para el cribado prenatal de aneuploidías. Sería muy recomendable establecer una estrategia nacional de cribado prenatal, con evaluación periódica de los indicadores y procedimientos diagnósticos, supervisada por las administraciones sanitarias. Los protocolos deberían ser revisados periódicamente para adaptarse a nuevas tecnologías coste-efectivas.

## Supplementary Material

Supplementary Material Details

Supplementary Material Details

Supplementary Material Details

## Material Suplementario

La versión en línea de este artículo ofrece material suplementario (https://doi.org/10.1515/almed-2019-0040).

## References

[1] (2002). Ley básica reguladora de la autonomía del paciente y de derechos y obligaciones en materia de información y documentación clínica.

[2] Palomaki GE, Lee JE, Canick JA, McDowell GA, Donnenfeld AE, For the ACMG Laboratory Quality Assurance Committee (2009). Technical standards and guidelines: prenatal screening for down syndrome that includes first-trimester biochemistry and/or ultrasound measurements. Genet Med.

[3] Public Health England (2107). Programme specific operating model for quality assurance of antenatal and newborn screening programmes.

[4] FMF Certification of Biochemical Laboratories (2020). Disponible en:.

[5] Spencer K (2005). First trimester maternal serum screening for Down's syndrome: an evaluation of the DPC Immulite 2000 free beta-hCG and pregnancy-associated plasma protein-A assays. Ann Clin Biochem.

[6] Benn PA, Collins R (2001). Evaluation of effect of analytical imprecision in maternal serum screening for Down's syndrome. Ann Clin Biochem.

[7] NHS Fetal Anomaly Screening Programme – Screening for Down’s, Edward’s and Patau’s Syndromes (Trisomies 21, 18 & 13) (2020). NHS public health functions agreement 2018–2019.

[8] Cuckle HS, Wald NJ, Thompson SG (1987). Estimating a woman's risk of having a pregnancy associated wih Down's syndrome using her age and serum alpha-fetoprotein level. Br J Obstet Gynecol.

[9] Chitayat D, Langlois S, Wilson RD (2011). Prenatal screening for fetal aneuploidy in singleton pregnancies. J Obstet Gynaecol Can.

[10] NHS (2020). National Down's Syndrome Screening Programme for England. Down's syndrome screening: risk calculation software requirements.

[11] Palomaki GE, Bradley LA, McDowell GA, Down Syndrome Working Group, ACMG Laboratory Quality Assurance Committee (2005). Technical standards and guidelines: prenatal screening for down syndrome. Gen Med.

[12] Salomon LJ, Alfirevic Z, Bilardo CM, Chalouhi GE, Ghi T, Kagan KO (2013). ISUOG practice guidelines: performance of first-trimester fetal ultrasound scan. Ultrasound Obstet Gynecol.

[13] (2020). GAP Exploración ecográfica del primer trimestre 2015.

[14] (2020). GAP Cribado y Diagnóstico precoz de anomalías genéticas 2018.

[15] Cuckle H, Platt LD, Thornburg LL, Bromley B, Fuchs K, Abuhamad A (2015). Nuchal Translucency Quality Review (NTQR) program: first one and half million results. Ultrasound Obstet Gynecol.

[16] NHS, ISUOG-Salomon 2013 (2020). NHS Fetal Screening Programme (NHS FASP). Recomended criteria for measurement of fetal crown rump length (CRL) as part of combined screening for Trisomy 21 within the NHS in England.

[17] Fries N, Althuser M, Fontanges M, Talmant C, Jouk PS, Tindel M (2007). Quality control of an image-scoring method for nuchal translucency ultrasonography. Am J Obstet Gynecol.

[18] Wanyonyi SZ, Napolitano R, Ohuma EO, Salomon LJ, Papageorghiou AT (2014). Image-scoring system for crown-rump length measurement. Ultrasound Obstet Gynecol.

[19] Dhombres F, Roux N, Friszer S, Bessis R, Khoshnood B, Jouannic JM (2016). Relation between the quality of the ultrasound image acquisition and the precision of the measurement of the crown-rump length in the late first trimester: what are the consequences?. Eur J Obstet Gynecol Reprod Biol.

[20] Sabria J, Guirado L, Miro I, Gomez-Roig MD, Borrell A (2017). Crown-rump length audit plots with the use of operator-specific PAPP-A and beta-hCG median MoM. Prenat Diagn.

[21] Cuckle H (2010). Monitoring quality control of nuchal translucency. Clin Lab Med.

[22] Fetal Medicine Foundation (2020). Disponible en.

[23] Herman A, Dreazen E, Maymon R, Tovbin Y, Bukovsky I, Weinraub Z (1999). Implementation of nuchal translucency image-scoring method during ongoing audit. Ultrasound Obstet Gynecol.

[24] Herman A, Maymon R, Dreazen E, Caspi E, Bukovsky I, Weinraub Z (1998). Nuchal translucency audit: a novel image-scoring method. Ultrasound Obstet Gynecol.

[25] Snijders RJ, Thom EA, Zachary JM, Platt LD, Greene N, Jackson LG (2002). First-trimester trisomy screening: nuchal translucency measurement training and quality assurance to correct and unify technique. Ultrasound Obstet Gynecol.

[26] Palomaki GE, Lee JE, Canick JA, McDowell GA, Donnenfeld AE, ACMG Laboratory Quality Assurance Committee (2009). Technical standards and guidelines: prenatal screening for Down syndrome that includes first-trimester biochemistry and/or ultrasound measurements. Genet Med.

[27] Malone FD, Canick JA, Ball RH, Nyberg DA, Comstock CH, Bukowski R (2005). First-trimester or second-trimester screening, or both, for Down's syndrome. N Engl J Med.

[28] Palomaki GE, Neveux LM, Donnenfeld A, Lee JE, McDowell G, Canick JA (2008). Quality assessment of routine nuchal translucency measurements: a North American laboratory perspective. Genet Med.

[29] Biau DJ, Porcher R, Salomon LJ (2008). CUSUM: a tool for ongoing assessment of performance. Ultrasound Obstet Gynecol.

[30] Sabria J, Barcelo-Vidal C, Arigita M, Jimenez JM, Puerto B, Borrell A (2011). The CUSUM test applied in prospective nuchal translucency quality review. Ultrasound Obstet Gynecol.

[31] Hynek M, Smetanova D, Stejskal D, Zvarova J (2014). Exponentially weighted moving average chart as a suitable tool for nuchal translucency quality review. Prenat Diagn.

[32] Chang WR, McLean IP (2006). CUSUM: a tool for early feedback about performance?. BMC Med Res Methodol.

[33] Grömminger S, Erkan S, Schöck U, Stangier K, Bonnet J, Schloo R (2015). The influence of low molecular weight heparin medication on plasma DNA in pregnant women. Prenat Diagn.

[34] Ashoor G, Syngelaki A, Poon LC, Rezende JC, Nicolaides KH (2012). Fetal fraction in maternal plasma cell-free DNA at 11–13 weeks' gestation: relation to maternal and fetal characteristics. Obstet Gynecol.

[35] Gil MM, Accurti V, Santacruz B, Plana MN, Nicolaides KH (2017). Analysis of cell-free DNA in maternal blood in screening for aneuploidies: updated meta-analysis. Ultrasound Obstet Gynecol.

[36] Grömminger S, Yagmur E, Erkan S, Nagy S, Schöck U, Bonnet J (2014). Fetal aneuploidy detection by cell-free DNA sequencing for multiple pregnancies and quality issues with vanishing twins. J Clin Med.

[37] Wong D, Moturi S, Angkachatchai V, Mueller R, DeSantis G, van den Boom D (2013). Optimizing blood collection, transport and storage conditions for cell free DNA increases access to prenatal testing. Clin Biochem.

[38] Vermeesch JR, Voet T, Devriendt K (2016). Prenatal and pre-implantation genetic diagnosis. Nat Rev Genet.

[39] Gregg AR, Skotko BG, Benkendorf JL, Monaghan KG, Bajaj K, Best RG (2016). Noninvasive prenatal screening for fetal aneuploidy, 2016 update: a position statement of the American College of Medical Genetics and Genomics. Genet Med.

[40] Wijnberger LDE, van der Schouw YT, Christiaens GCML (2000). Learning in medicine: chorionic villus sampling. Prenat Diagn.

[41] Mungen E, Tutuncu L, Muhcu M, Yergok YK (2006). Pregnancy outcome following second-trimester amniocentesis: a case-control study. Am J Perinatol.

[42] Alfirevic Z (2009). Who should be allowed to perform amniocentesis and chorionic villus sampling?. Ultrasound Obstet Gynecol.

[43] Tabor A, Vestergaard CHF, Lidegaard O (2009). Fetal loss rate after chorionic villus sampling and amniocentesis: an 11-year national registry study. Ultrasound Obstet Gynecol.

[44] McWeeney DT, Schwendemann WD, Nitsche JF, Rose CH, Davies NP, Watson WJ (2012). Transabdominal and transcervical chorionic villus sampling models to teach maternal–fetal medicine fellows. Am J Perinatol.

[45] Nizard J, Duyme M, Ville Y (2002). Teaching ultrasound-guided invasive procedures in fetal medicine: learning curves with and without an electronic guidance system. Ultrasound Obstet Gynecol.

[46] Karasahin E, Alanbay I, Ercan M, Yenen MC, Dede M, Baser I (2009). Simple, cheap, practical and efficient amniocentesis training model made with materials found in every obstetrics clinic. Prenat Diagn.

[47] Wax JR, Cartin A, Pinette MG (2012). The birds and the beans: a low-fidelity simulator for chorionic villus sampling skill acquisition. J Ultrasound Med.

[48] Pittini R, Oepkes D, Macrury K, Reznick R, Beyene J, Windrim R (2002). Teaching invasive perinatal procedures: assessment of a high fidelity simulator-based curriculum. Ultrasound Obstet Gynecol.

[49] Khurshid N, Trampe B, Heiser T, Birkeland L, Duris E, Stewart K (2014). Impact of an amniocentesis simulation curriculum for training in MFM fellowship program. Am J Obstet Gynecol.

[50] Royal College of Obstetricians and Gynaecologists (2010). Amniocentesis and chorionic villus sampling. Green-top Guideline No. 8.

[51] Royal Australian and New Zealand College of Obstetricians and Gynaecologists (2020). Certification in maternal fetal medicine training program handbook.

[52] Saura R, Gauthier B, Taine L, Wen ZQ, Horovitz J, Roux D (1994). Operator experience and fetal loss rate in transabdominal CVS. Prenat Diagn.

[53] ISUOG Practice Guidelines (2016). Invasive procedures for prenatal diagnosis. Ultrasound Obstet Gynecol.

[54] Halliday JL, Sheffield LJ, Danks D, Lumley J (1990). Complete follow-up in assessing fetal losses after chorionic villus sampling. Lancet.

[55] Salomon LJ, Sotiriadis A, Wulff CB, Odibos A, Akolekar R (2019). Risk of miscarriage following amniocentesis or chorionic villus sampling: systematic review of literature and updated meta-analysis. Ultrasound Obstet Gynecol.

[56] Cherry AM, Akkari YM, Barr KM, Kearney HM, Rose NC, South ST (2017). Diagnostic cytogenetic testing following positive noninvasive prenatal screening results: a clinical laboratory practice resource of the American College of Medical Genetics and Genomics (ACMG). Genet Med.

[57] Association for clinical cytogenetics (2009). Prenatal diagnosis best practice guidelines.

[58] Suela J, López-Expósito I, Querejeta ME, Martorell R, Cuatrecasas E, Armengol L (2017). Recommendations for the use of microarrays in prenatal diagnosis. Med Clin (Barc).

