# Supplementary material for: Cribado y diagnóstico prenatal de anomalías genéticas: recomendaciones de consenso SEGO, SEQCML, AEDP
Source: Adv Lab Med. 2020 Jun 22;1(3):20190040. [Article in Spanish] doi: 10.1515/almed-2019-0040 (PMC10197968; doi:10.1515/almed-2019-0040)
Supplement: Supplementary file 1 — Supplementary Material Details [file j_almed-2019-0040_suppl1.doc]

**Tabla suplementaria 1: Indicadores de evaluación del proceso de captación y marcadores bioquímicos de un cribado combinado (CC) de primer trimestre**

| **Indicadores de evaluación de captación y proceso bioquímico** | | | |
| --- | --- | --- | --- |
| **Numerador** | **Denominador** | **Indicador en %** | **Estándar** |
| Gestantes captadas para el cribado combinado (CC) | Total de gestantes | Participación en el CC | >80% |
| Formularios de solicitud de cribado con datos demográficos incompletos | Gestantes captadas para el CC | % datos demográficos incompletos | <5% |
| Formularios de solicitud de cribado con datos necesarios para el cálculo incompletos | Gestantes captadas para el CC | % datos necesarios para el cálculo incompletos | 0% (no se validarán pruebas bioquímicas hasta obtener los datos necesarios) |
| Condiciones de transporte y conservación de muestras para cribado | - | - | 100% en las condiciones preanalíticas establecidas (requisito) |
| Gestantes con cribado bioquímico en primer trimestre, sin medida de TN | Total de gestantes captadas para CC | % CC incompleto | <1% sin causa justificada, como aborto, interrupción legal del embarazo, defectos del tubo neural… |
| Gestantes cribadas con test bioquímico, por semana de gestación (SG) | Total de gestantes con CC completo | Captación por SG | Marcadores bioquímicos analizados fuera de SG óptima por error de datación (discordancia de fechas)  2 pasos: <10%  1 paso: <1% |
| Gestantes con CC completo | Partos | Estimación de Cobertura CC | >95% |
| Error total riesgo bioquímico en el periodo evaluado | - | - | <10% |
| Mediana de los MdM de cada marcador bioquímico en el periodo evaluado | - | - | 0,9 – 1,1 |

| **Otros indicadores globales del cribado combinado (CC)** | | | |
| --- | --- | --- | --- |
| **Numerador** | **Denominador** | **Indicador en %** | **Estándar** |
| Nº cribados con riesgo alto de T21/18 en primer trimestre: especificar punto de corte | Total de cribados primer trimestre | - | Estimación riesgo alto en el primer trimestre |
| Nº cribados completos, por SG de Ecografía TN | Total de gestantes con CC completo | % cribados según SG de Ecografía TN | - |
| Gestantes con desenlace conocido | Total de gestantes captadas para CC | % casos con seguimiento hasta el parto | >95% |
| Gestantes con afectado conocido | Total de gestantes captadas para CC | % casos para evaluación | >95% |
| Gestantes con riesgo alto y feto afecto: Verdaderos Positivos (VP) | Total de gestantes con fetos afectos | - | Estimación de la Sensibilidad |
| Gestantes con riesgo bajo y fetos no afectos: Verdaderos Negativos (VN) | Total de gestantes con fetos no afectos | - | Estimación de la Especificidad |
| Gestantes con riesgo alto y feto afecto (VP) | Total de gestantes con riesgo alto | - | Estimación de valor predictivo positivo |
| Casos T21,18,13 diagnosticados | Total de gestantes con fetos afectos | - | Estimación de Prevalencia |
