# Supplementary material for: Cribado y diagnóstico prenatal de anomalías genéticas: recomendaciones de consenso SEGO, SEQCML, AEDP
Source: Adv Lab Med. 2020 Jun 22;1(3):20190040. [Article in Spanish] doi: 10.1515/almed-2019-0040 (PMC10197968; doi:10.1515/almed-2019-0040)
Supplement: Supplementary file 2 — Supplementary Material Details [file j_almed-2019-0040_suppl2.doc]

**Tabla suplementaria 2. Propuesta de registro para evaluación de indicadores del test ADNlc (n, %)**

| - Pruebas realizadas - antes de los estudios del primer trimestre - en embarazos con alto riesgo - en embarazos con TRA (reproducción asistida) - en embarazos gemelares - sin indicación de acuerdo con los protocolos actuales (SEGO) - en embarazadas con índice de masa corporal > 35 |
| --- |
| - Pruebas sin resultado final |
| - ALTOS RIESGOS totales y desglosados |
| - Tasa de FP en T21, T18 y T13. Incluir otras (ej: sexo) si se considera relevante |
| - Tasa de VP |
| - FALSOS NEGATIVOS y posibles causas |
