# Supplementary material for: Cribado y diagnóstico prenatal de anomalías genéticas: recomendaciones de consenso SEGO, SEQCML, AEDP
Source: Adv Lab Med. 2020 Jun 22;1(3):20190040. [Article in Spanish] doi: 10.1515/almed-2019-0040 (PMC10197968; doi:10.1515/almed-2019-0040)
Supplement: Supplementary file 3 — Supplementary Material Details [file j_almed-2019-0040_suppl3.doc]

**Tabla suplementaria 3. Propuesta de indicadores de evaluación para las pruebas invasivas**

*Pé* Pérdidas globales post biopsia corial (PBC), no sólo las atribuibles a la técnica

| **Indicadores aplicables a las pruebas invasivas derivadas del cribado combinado** | | | |
| --- | --- | --- | --- |
| **Numerador** | **Denominador** | **Indicador** | **Estándar** |
| Pruebas invasivas con riesgo alto | Gestantes con riesgo alto | % prueba invasiva con indicación de riesgo | - |
| Pérdidas fetales post biopsia corial (PBC)* | Número biopsias coriales realizadas por alto riesgo anomalía genética | % pérdida fetal global PBC | <1,5% |
| Pérdidas fetales post amniocentesis (PA)** | Número de amniocentesis realizadas por alto riesgo de anomalía genética | % pérdida fetal global PA | <1% |
| Pruebas invasivas sin indicación de riesgo | Gestantes con riesgo bajo | % prueba invasiva sin indicación de riesgo | <0,5% |

** Pérdidas globales post amniocentesis (PA), no sólo las atribuibles a la técnica
